# Regulation of Compound Leaf Development

**DOI:** 10.3390/plants3010001

**Published:** 2013-12-19

**Authors:** Yuan Wang, Rujin Chen

**Affiliations:** Plant Biology Division, Samuel Roberts Noble Foundation, Ardmore, OK 73401, USA; E-Mail: ywang@noble.org

**Keywords:** compound leaf development, gene regulation, hormones, tomato, *Cardamine hirsuta*, *Medicago truncatula*

## Abstract

Leaf morphology is one of the most variable, yet inheritable, traits in the plant kingdom. How plants develop a variety of forms and shapes is a major biological question. Here, we discuss some recent progress in understanding the development of compound or dissected leaves in model species, such as tomato (*Solanum lycopersicum*), *Cardamine hirsuta* and *Medicago truncatula*, with an emphasis on recent discoveries in legumes. We also discuss progress in gene regulations and hormonal actions in compound leaf development. These studies facilitate our understanding of the underlying regulatory mechanisms and put forward a prospective in compound leaf studies.

## 1. Introduction

Plant leaves are the primary photosynthetic organs responsible for the conversion of carbon dioxide (CO_2_) and water (H_2_O) to carbohydrate (C_6_H_12_O_6_) and oxygen (O_2_) using solar energy. As such, it provides the fixed carbon for plant growth and atmospheric oxygen essential for aerobic lives. Together, photosynthetic organisms, including plants, algae and some bacteria, capture approximately 100 terawatts (10^12^ watts) of solar energy and convert about 100–150 petagrams (10^15^ grams) of carbon into biomass per year [1,2]. The photosynthetic structures of the earliest vascular plants are believed to be branched axes, which evolved into three-dimensional lateral branch systems identified as the earliest leaves in the fossil record [3]. From ferns to angiosperms, enormous variations in leaf forms have been evolved. Conceptually, leaf forms can be broadly grouped into two categories: simple and compound or dissected. A simple leaf consists of a single undivided blade with an entire, serrated or lobed margin, and a compound leaf consists of multiple blades, known as leaflets, organized as pinnate, palmate or higher-ordered structures [4]. Both developmental and environmental factors influence leaf morphogenesis [5]. Many plants exhibit consistent leaf forms, although variations in the leaf morphology, called heteroblasty, often occur during the course of plant growth [6]. Different leaf forms can be found in closely-related plants, such as *Arabidopsis thaliana*, with simple leaves, and *Cardamine hirsuta*, with compound leaves [7]. In addition, different leaf forms can be found in the same plant, such as green ash (*Fraxinus pennsylvanica* var. *lanceolata*) and *Medicago truncatula*, in which leaf forms change from simple to compound along the shoot axis [8,9].

The diverse leaf shape and form variation seen in extant plants may reflect natural selection on the function of leaves as the primary photosynthetic organ critical to plant growth and survival [10,11,12]. Studies of leaf evolution suggest that compound leaves evolved independently several times during plant evolution [13,14]. Consistent with this, both conserved and distinct molecular mechanisms underlying the development of different leaf forms have been uncovered in different lineages [13,15]. 

In earlier studies, numerous leaf form mutants have been isolated and characterized in both simple- and compound-leafed species, such as maize (*Zea may*), snapdragon (*Antirrhinum majus*), *Arabidopsis* (*A. thaliana*), tobacco (*Nicotiana tabacum*), pea (*Pisum sativum*), tomato (*Solanum lycopersicum*), *C. hirsuta* and *M. truncatula*. In the last few decades, many genetic loci responsible for some of the classic leaf form mutants have been cloned, unveiling the nature of genes and the associated regulatory mechanisms underlying leaf development in diverse species. These provide opportunities to compare the regulatory mechanisms for compound- and simple-leaf development and provide new insights on leaf form evolution.

## 2. Compound Leaf Development

Plant leaves, either simple or compound, initiate as peg-like structures from the flanks of the shoot apical meristem (SAM), a pluripotent structure capable of self-renewal. Studies have shown that class I knotted-like homeodomain transcription factors, KNOXI proteins, are required for the maintenance of the meristematic activity of the SAM [16,17,18]. Leaf initiation requires downregulation of *KNOXI* genes at the incipient sites of leaf primordia (P0, P for plastochron). In species with simple leaves, downregulation of *KNOXI* genes is permanent, whereas in most compound-leafed eudicot species, including tomato and *C. hirsuta*, *KNOXI* genes are reactivated in leaf primordia after initial downregulation to initiate leaf development [16,17]. In C. hirsuta and tomato, downregulation of *KNOXI* genes result in compound leaves with reduced leaflets [7,19,20]. On the other hand, ectopic overexpression of *KNOXI* genes in the tomato dominant mutants, *Mouse Ears* (*me*) and *Curl* (*Cu*) [21,22], or ectopic expression of *KNOXI* genes in tomato plants [23,24] results in a dramatic increase in leaf complexity. 

Leaf development can be divided into three distinct stages, initiation, primary morphogenesis and secondary morphogenesis or histogenesis [20,25,26]. During primary morphogenesis of compound leaves, leaf primordia after initiating from the flanks of the SAM expand laterally and develop secondary structures from specific meristematic regions at the leaf margin, termed marginal blastozones [27]. During secondary morphogenesis, leaves undergo extensive cell expansion and differentiation. Depending on the timing of expression, overexpression of *KNOXI* genes in tomato results in different effects on leaf shapes, consistent with a context-dependent role of *KNOXI* genes in promoting meristematic activity and preventing maturation [20]. 

In contrast to KNOXI, the CINCINNATA (CIN)-like TCP transcription factor, LANCEOLATE (LA), restricts the activity of the leaf marginal blastozone in tomato. *La-2*, a dominant *LA* mutant, has simple leaves with uniform blades; whereas overexpression of miR319, which targets and downregulates several *LA*-like genes, results in compound leaves with indeterminate growth [28,29,30]. 

The MYB domain protein encoded by the *ARP* gene, *ASYMMETRIC LEAF1* (*AS1*) in *Arabidopsis* (*A. thaliana*), has been shown to act together with the LATERAL ORGAN BOUNDARY (LOB) domain protein, AS2, to exclude expression of *KNOXI* genes in incipient leaf primordia. *SHOOT APICAL MERISTEMLESS* (*STM*), a *KNOXI* gene, acts, in turn, to exclude *AS1* expression in the SAM. These antagonistic interactions are not only important for the maintenance of the meristematic activity of the SAM, but also for the development of leaf primordia in *Arabidopsis*. In compound-leafed *C. hirsuta*, mutations in the *AS1* gene result in ectopic expression of *STM* and compound leaves with an increased order of complexity [7,31]. These results indicate that the MYB domain protein, ARP, also plays a role in compound leaf development by restricting the expression of *KNOXI* genes. However, the regulatory relationship between *ARP* and *KNOXI* genes is more complex in tomato [32]. It has been shown that downregulation of the tomato *PHAN* gene, *SlPHAN*, results in radialized or peltately-palmate compound leaves and a loss of the typical pinnate compound leaves of wild-type plants [33]. 

## 3. Auxin Plays a Critical Role in the Initiation, Patterning and Morphogenesis of Compound Leaves

The plant hormone, auxin, plays a critical role in diverse plant growth and developmental processes, such as organogenesis, vascular tissue differentiation and tropisms. At the periphery of the SAM, convergence of the auxin efflux carrier PIN1 protein in the L1 layer mediates the formation of auxin maxima and marks the incipient sites of leaf primordia [34]. Furthermore, the spatiotemporal distribution of the convergence points and associated auxin maxima at the flanks of the SAM determines phyllotactic arrangements of leaves [34,35].

In compound-leafed species, including tomato, *C. hirsuta* and *M. truncatula*, auxin maxima and convergence points of PIN1 proteins coincide with leaf and leaflet initiation [31,36,37]. Whole-plant treatments with exogenous auxin lead to ectopic blade outgrowth along the leaf rachis [31], and intriguingly, local applications of auxin induce leaflet initiation [31,36]. On the other hand, treatments with auxin transport inhibitors or auxin antagonists result in simplification of leaf forms in pea, tomato and *C. hirsuta* [31,36,38,39]. Consistent with physiological studies, *C. hirsuta* *pin1* mutants develop simple leaves [31]. Interestingly, loss-of-function mutations of the *Medicago* *PIN10* gene (*MtPIN10*), the *PIN1* ortholog, result in compound leaves with various numbers of leaflets, consistent with fusion of leaf primordia at various developmental stages [40], and with smooth leaf margins [37,40]. Collectively, these studies uncover that auxin maxima generated by convergence points of auxin efflux transporters direct leaf and leaflet initiation. In addition, it has been shown that auxin is required for KNOXI-mediated leaf dissection in tomato [31]. Together, these studies support that the auxin activity maxima are one of the earliest molecular events for leaflet initiation. Previous studies have shown that auxin signaling and response play a role in leaf dissection. It is known that auxin responsive Aux/IAA proteins negatively regulate AUXIN RESPONSE FACTOR (ARF) protein activities and, thereby, repress auxin response [41,42]. Downregulation of *SlIAA9* in antisense transgenic plants results in simplified leaves, phenocopying a spontaneous tomato mutant, *entire* (*e*), caused by a single base deletion in the *SlIAA9* gene [43,44]. *E*/*SlIAA9* is expressed in the leaf marginal blastozone and vascular tissues [36,45] and functions to restrict lamina outgrowth between leaflets [36,45]. SlARF10, a positive regulator of auxin response, functions as a repressor of lamina outgrowth in tomato [46], further supporting a role of auxin response in leaf blade outgrowth. Recently, E/SlIAA9 has been shown to directly interact with the auxin receptors, SlTIR1 and SlAFB6, and is subject to degradation by the ubiquitin 26S proteasome SCF^TIR1/AFB^ in an auxin-dependent manner, and these resemble the Aux/IAA proteins in *A. thaliana* [45,47,48]. Similarly, tomato *goblet* (*gob*) mutants of the *NO APICAL MERISTEM* (*NAM*)/*CUP-SHAPED COTYLEDON* (*CUC*) gene also develop primarily primary leaflets [49]. In both *e* and *gob* mutants, the auxin signal, as shown by the DR5 auxin response sensor, expands to the entire leaf margin [45]. Inhibition of auxin transport and activity suppresses the *GOB* overexpression phenotype [45]. These observations are consistent with the hypothesis that proper leaflet initiation and separation requires distinct boundaries between regions of lamina growth and adjacent regions of growth suppression [45]. 

## 4. Gibberellic Acid and Cytokinin in Compound Leaf Development

Gibberellic acid (GA) and cytokinin (CK) are both growth-promoting hormones, but they function through different mechanisms: CK primarily promotes cell division, and GA regulates cell expansion and differentiation. Interestingly, in many different developmental processes, they act antagonistically, and these antagonistic interactions may occur during biosynthesis, catabolism or signaling [50,51,52,53,54]. In the SAM, CK suppresses GA-mediated cell differentiation to maintain the indeterminacy of the meristem, whereas GA represses CK-mediated cell division at the sites of leaf initiation, allowing leaf organogenesis [55]. It is known that KNOXI proteins promote and maintain the activity of the SAM through activating the biosynthesis of CKs, which, in turn, represses GA activities [54,56,57]. 

The involvement of GA in dissected leaf development was proposed as early as the 1950s. Recent physiological, genetic and molecular studies support and further refine the earlier studies. It has been observed that exogenous applications of GA to developing leaves lead to a simplified leaf form and smooth leaf margins in tomato [56,58,59,60]. 2-oxoglutarate-dependent dioxygenase, GA20 oxidase (GA20ox), is a key enzyme in the GA biosynthesis [61,62], whereas GA2 oxidase (GA2ox) deactivates bioactive GAs [63,64]. These genes coordinately regulate levels of bioactive GAs in plants and, thus, determine the homeostasis of GAs. Overexpression of *SlGA2ox4* in tomato leaves results in an increased leaf complexity [29]. These observations support that dissected leaf morphogenesis is sensitive to alterations in the GA activity in tomato.

GA regulates plant growth and development by suppressing the growth repressors, DELLA proteins [65,66]. A mutation in *PROCERA* (*PRO*), encoding a DELLA protein, results in simplified leaves and smooth leaf margins, supporting that the DELLA-dependent GA signaling negatively regulates leaflet initiation during early leaf development in tomato [67,68,69,70,71,72]. It has been postulated that DELLA proteins regulate the leaflet number by defining the appropriate timing for leaflet initiation. 

Up until now, it is still not fully understood how GA participates in dissected leaf development. Experimental evidence supports that GA signaling partly mediates the function of KNOXI proteins. On the other hand, KNOXI proteins directly repress the expression of GA20ox and activate the expression of GA2ox in both simple- and compound-leafed species [52,54,56,73]. Thus, KNOXI proteins antagonize the GA activity. Genetic analyses indicate that altered GA homeostasis or signaling modulates *KNOXI* misexpression phenotypes in *A. thaliana*. Moreover, exogenous GA applications or the *pro* mutations suppress the super-compound leaf phenotype in the tomato dominant mutant, *Mouse ears* (*Me*), in which the tomato *KNOXI* gene, *Tkn2*, is misexpressed [56]. Although reducing the GA activity itself is not sufficient to increase the leaflet number in wild-type tomato, it enhances the competence to develop more leaflets of *Me* or *Curl* (*Cu*) mutants [54]. The recessive loss-of-function *clausa* (*clau*) mutant, which fails to delimit *KNOXI* expression domains, displays elevated and broadened *KNOXI* expression and increased leaf complexity. Exogenous GA applications or *pro* mutations suppress the compound leaf phenotype of the *clau* mutant, whereas mutations that lead to GA deficiency act conversely, suggesting that GA signaling acts downstream of the endogenous *KNOXI* pathway in tomato [54]. Thus, KNOXI proteins function in the maintenance of the SAM and promote compound leaf development partly by repressing GA homeostasis and response.

GA signaling is considered to partly mediate the LANCEOLATE (LA) activity in tomato. As discussed earlier, the maintenance of the morphogenetic activity of the leaf marginal blastozone required for the elaboration of leaflets involves the positive regulators, KNOXI proteins, and the negative regulator, LA, in tomato. *LA* promotes determinate growth and is negatively regulated by miR319 [30]. Interestingly, GA-treated wild-type plants resemble the *La-2* mutant in leaf complexity, leaf margin serration, hypocotyl length and anthocyanin content [29]. Exogenous GA applications or constitutive GA responses conditioned by the *pro* mutation suppress the indeterminate leaf growth caused by overexpression of miR319. In addition, the long hypocotyl phenotype of the *La-2* mutant is suppressed by mutations that lead to GA deficiencies or by applications of GA biosynthesis inhibitors, further supporting that the gain-of-function phenotype of *La-2* is mediated by an increased GA activity [29]. Consistent with this, genes involved in the GA metabolism or response is differentially regulated in the *La-2* mutant. Of particular interest among the differentially-regulated genes is *SlGA2ox4*. Overexpression of *SlGA2ox4* partially suppresses the simple-leaf phenotype of *La-2*. The relationship between the GA pathway and LA function appears to be evolutionarily conserved, at least in *A. thaliana* and tomato [29].

CK coordinates a wide range of plant developmental processes [55,74]. During shoot development, CK positively regulates the size and activity of the SAM [75,76,77,78,79,80,81]. Interactions between genes related to CK biosynthesis and response and genes required for the SAM maintenance exist at various levels. As discussed, KNOXI proteins activate CK biosynthesis [54,57]. In turn, CK regulates expression and activity of *WUSCHEL*, a key regulator of the meristem [77,82]. 

Recently, it has been reported that cytokinin regulates dissected leaf morphogenesis in tomato [83]. Manipulation of the endogenous CK level by ectopic expression of the CK metabolic genes, *AtIPT7* and *AtCKX3*, in developing leaf primordia alters leaf complexity in tomato. This has been attributed to CK regulation of the window of morphogenetic activity at leaf margins. Consistent with this, an extended CK response in leaf margins, as indicated by the upregulation of the CK response markers, RRs, has been shown to correlate with an increased leaf complexity [83]. Both local applications of auxin to developing leaf primordia and mutations in *SlIAA9* encoding an auxin response repressor suppress the *AtIPT7*-induced supercompound leaf phenotype, suggesting that CK regulation of dissected leaf morphogenesis requires a localized auxin response. Interestingly, auxin transport and distribution do not appear to be affected by alterations of the CK level. A decrease in the endogenous CK level suppresses and an increase in the CK level compensates for the effect of KNOXI on leaf complexity, supporting the notion that CK acts downstream of KNOXI proteins in dissected leaf development, similar to their interactions in the SAM [83]. Thus, CK positively regulates leaf complexity in tomato. 

The antagonistic interactions between GA and CK are dependent on the GA/CK ratio rather than the levels of each hormone, at least in tomato [60]. Intriguingly, the interactions between GA and CK during compound leaf development do not appear to be dependent on the DELLA protein. Together, these results suggest that GA and CK act downstream of the KNOXI and LA proteins to fine-tune the morphogenetic window and, thereby, regulate compound leaf development in tomato.

## 5. Compound Leaf Development in Legumes

KNOXI proteins maintain the indeterminacy required for compound leaf development in most eudicot species, including tomato and *C. hirsuta*. However, in some leguminous plants (Fabaceae) that belong to the inverted repeat lacking clade (IRLC), *UNIFOLIATA* (*UNI*) in pea and *SINGLE LEAFLET1* (*SGL1*) in *M. truncatula*, encoding legume FLORICAULA (FLO)/LEAFY (LFY)-type transcription factors, have been shown to play a similar role in compound leaf development as *KNOXI* genes in tomato and *C. hirsuta* [9,84]. This is mainly due to the following observations. Firstly, *KNOXI* genes and encoded proteins are not expressed in leaves and leaf primordia and, therefore, do not likely play a role in compound leaf development in pea and *Medicago* [9,13,85,86], although a conflicting report shows that a low level of expression of *KNOXI* genes could be detected in developing leaves in pea and *M. truncatula* [87]. Secondly, pea *uni* and *Medicago* *sgl1* mutants exhibit simple leaves and *Medicago* *palmate-like pentafoliata* (*palm1*) mutants, in which *SGL1* is ectopically upregulated, exhibit an increased complexity of compound leaves [88]. Thirdly, a loss-of-function mutant of the *Medicago* *KNOXI* gene, *BREVIPEDICELLUS* (*MtBP*), does not exhibit compound leaf defects [89].

*M. truncatula* has been selected as a model species for genetics and genomics studies in legumes, because it has a relatively small and diploid genome (~600 Mb), a short lifecycle and an available genetic transformation system. Recently, its genome has been sequenced [90]. Also available are several comprehensive mutant collections, including the ethyl methane sulfonate (EMS) collection [91], the fast neutron bombardment (FNB) deletion mutant collection [86,92,93] and the insertion mutant collections [94,95,96,97,98]. These mutant collections are excellent resources for forward and reverse genetic studies. 

**Figure 1:**
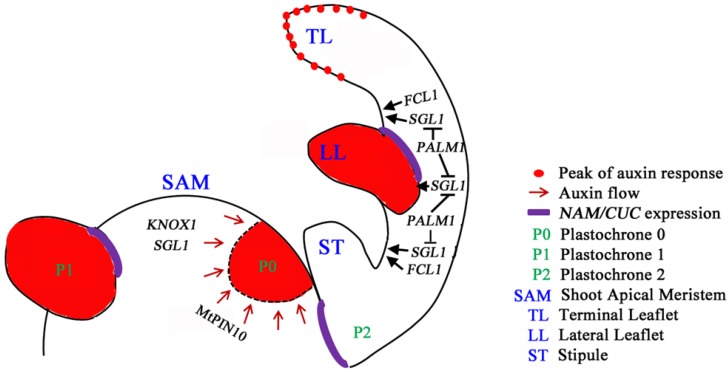
Compound leaf development in inverted repeat lacking clade (IRLC) legumes. Based on the understanding of compound leaf development in *Medicago truncatula* and pea (*Pisum sativa*) belonging to the IRLC clade of legumes, a genetic model that governs compound leaf development is proposed. The class I homeodomain transcription factor *KNOXI* genes are expressed in the shoot apical meristem (SAM), but their expression is excluded from incipient sites of leaf primordia at the periphery of SAM and subsequent leaf primordia. Loss-of-function mutants of the *FLO/LFY*/*UNI* ortholog, *SGL1*, exhibit simple leaves with reduced petioles, consistent with the role of *SGL1/UNI* in lateral leaflet initiation and leaf proximodistal axis development. The C2H2 zinc finger transcription factor, PALM1, directly represses the expression of *SGL1* to regulate its spatiotemporal expression domain. *FCL1*, encoding a class M KNOX protein, is required for the leaf proximodistal axis development and leaflet boundary formation. *FCL1* and *SGL1* act in parallel pathways, and both are required for leaf petiole development. *Medicago* auxin efflux PIN10 protein (MtPIN10), the *Arabidopsis* PIN1 ortholog, mediates auxin activity maxima that precede and are required for the initiation of leaf and leaflet primordia and leaflet serration at the distal leaflet margin. The *Medicago* *NAM*/*CUC* gene, *MtNAM*, functions in the development of lateral organ boundaries.

Similar to alfalfa (*M. sativa*) and soybean (*Glycine max*), *M. truncatula* plants exhibit trifoliate compound leaves. *Medicago* *single leaflet1* (*sgl1*) mutants fail to initiate lateral leaflets and exhibit only simple leaves, similar to pea *uni* mutants, and the mutant phenotypes are consistent with the role of SGL1/UNI in maintaining the morphogenetic activity at leaf margins to elaborate lateral leaflets (Figure 1) [9,84]. It has been shown that *SGL1* also plays a role in the proximal-distal axis development, because *sgl1* mutants exhibit significantly reduced petioles [9,86]. 

In contrast to the *sgl1* mutants with simple leaves, *Medicago* *palm1* mutants develop dissected leaves with five leaflets clustered at the leaf tip and increased petioles [88]. The appearance of two extra lateral leaflets occurs at the P3 stage of leaf primordia [88]. *PALM1* encodes a novel transcription factor with an *N*-terminal Cys(2)His(2) zinc finger DNA-binding domain [100] and a *C*-terminal EAR transcription repressor domain [88,100,101]. PALM1 binds to a specific sequence located in the promoter region of *SGL1* and negatively regulates the spatiotemporal expression of *SGL1*. Double mutant analysis shows that *SGL1* is required for the development of lateral leaflets in the *palm1* background. Thus, the intricate interactions between *PALM1* and *SGL1* determine the trifoliate leaf form in *M. truncatula* (Figure 1). Intriguingly, ectopic expression of *PALM1* completely suppresses leaf lobing phenotypes of *A. thaliana* plants that overexpress the *KNOXI* gene, *KNAT1*. This suggests that although KNOXI proteins are not associated with compound leaf development in *M. truncatula*, *PALM1* has the capacity to negatively regulate downstream targets or regulatory networks that are responsive to KNOXI activities. An extension of this hypothesis is that the negative regulation of KNOXI activities by *PALM1* orthologs may be operational in non-IRLC legumes, in which KNOXI proteins may play a role in leaf development. Phylogenetic analysis shows that *PALM1* homologous sequences exist in diverse species, ranging from closely-related legumes to lower land plants [101], suggesting an ancient origin and divergent function of *PALM1* in the development of different leaf forms. Interestingly, loss-of-function of *PALM1* also results in the loss of epicuticular wax crystals on the abaxial leaf surface, suggesting a role of *PALM1* in epicuticular wax biosynthesis and/or distribution [102]. 

Recently, a class M *KNOX* gene, *FUSED COMPOUND LEAF1* (*FCL1*) has been shown to play a positive role in boundary and proximodistal axis development of compound leaves in *M. truncatula* [86]. Positional cloning and genetic complementation tests reveal that *FCL1* encodes a class M KNOX protein, with a MEINOX domain, but without the DNA-binding homeodomain normally present in the canonical KNOXI proteins. Double mutant analyses show that *fcl1* is epistatic to *palm1*, and on the other hand, *sgl1* is epistatic to *fcl1* in leaf complexity; in terms of petiole development, *SGL1* and *FCL1* act additively. It is not yet clear how *FCL1* regulates compound leaf development in *M. truncatula*. Class M KNOX genes are also found in *Arabidopsis* and tomato [103,104]. It has been shown that the encoded proteins form heterodimers with BEL1-like homeodomain (BELL) proteins that themselves are KNOXI-interacting partners and, thereby, interfere with nuclear targeting and downstream regulatory networks of KNOXI-BELL complexes [103,104]. Although no loss-of-function mutants have been isolated from *A. thaliana* and tomato, ectopic expression of *KNATM-B* results in elongated petioles and narrow, shorter and serrated leaves in *Arabidopsis* [104]. In tomato, a semi-dominant *Petroselinum* (*Pts*) mutant that overexpresses the tomato class M *KNOX* gene, *PTS/TKD1*, due to a promoter mutation, exhibits a proliferation of compound leaves, affecting both primary and secondary leaflets [103]. In *M. truncatula*, the canonical *KNOXI* genes are not expressed in the leaf primordia of wild-type and *fcl1* mutants [86]. It is unlikely that FCL1 interferes with the function of the canonical KNOXI proteins during leaf development. However, FCL1 has been shown to interact in a yeast two-hybrid system with a subset of the *Arabidopsis* BELL homeodomain proteins, albeit with slightly different substrate specificities from KNATM-B [86]. It is plausible that FCL1 plays a role in compound leaf development through interactions with specific BELL proteins in *M. truncatula*. The *fcl1* mutant phenotypes suggest that FCL1 is required to maintain a window of organogenetic activities at leaf margins. Loss-of-function mutations of *FCL1* shorten the window of organogenetic activities and promote precocious leaf maturation, leading to leaflet fusion and clustering and a reduction in petiole length (Figure 1). 

*MtPIN10*, encoding an auxin efflux carrier protein orthologous to the *Arabidopsis* PIN1 protein, has been shown to play a key role in lateral organ development in *M. truncatula* [37,40]. *Medicago* *pin10* mutants (also named *smooth leaf margin1* or *slm1*) exhibit pleiotropic phenotypes, including an increased number of cotyledons, an increase in compound leaf complexity, smooth leaf margins, altered phyllotaxy and defective flower development. *Trans*-species genetic complementation studies demonstrate that MtPIN10 and *Arabidopsis* PIN1 are functional orthologs. Consistent with previous studies on PIN1 orthologs in compound-leafed species, MtPIN10/SLM1 mediates local auxin activity maxima in incipient sites of leaf primordia and leaflet serration in *M. truncatula* (Figure 1). The various compound leaf phenotypes observed in the *mtpin10* mutants have been attributed to a failure in separating leaf primordia at the flanks of the SAM, which leads to the fusion of leaf primordia at various developmental stages [40]. 

Boundary genes are required for the separation of leaflets. As discussed earlier, the *NAM/CUC* genes, from the large and evolutionarily conserved family of NAC transcription factor genes, specify organ boundaries in a diverse range of eudicot species [105]. The *M. truncatula* *NAM/CUC2* ortholog*, NO APICAL MERISTEM* (*MtNAM*), has been shown to exert a similar function as *NAM/CUC* genes in other species [106]*.* Besides the conserved function in organ separation, *MtNAM* has been shown to play a role in floral organ identity regulation.

## 6. Perspective and Biological Significance of Leaf Development Studies in *M. truncatula*

Because of the availability of a high-quality genome sequence, orthologous genes of key regulators of compound leaf development from other species can be, in most cases, identified in *M. truncatula*. Conservation and divergence can be further analyzed using protein coding and flanking sequences and synteny information across species. Reverse genetic screening is available to isolate mutant alleles of the orthologous genes, for phenotypic analysis. Genetic interactions between different compound leaf regulators can also be explored by double mutant analysis. These phylogenetic, genetic and functional studies are thus far used to dissect and compare gene regulatory networks controlling compound leaf development in *M. truncatula* and other species. On the other hand, the forward genetic approach has been used to isolate and characterize novel mutants with defects in leaf form, shape or patterning. Collectively, these ongoing studies will likely reveal new insight into novel regulators or the deployment of conserved regulators of compound leaf development. Recently, small RNAs, including microRNA and *trans*-acting small interfering RNA (*t*a-siRNA), known to play a role in plant growth, development and responses to the environment [107,108], have also been shown to regulate dissected leaf development [30,109,110,111]. It is foreseeable that the small RNA pathways will also be identified in forward or reverse genetic screens of compound leaf mutants in *M. truncatula*. 

Leguminous plants, belonging to the third largest family of flowering plants, include many important grain and forage crops, such as soybean (*Glycine max*), lentil (*Lens culinaris*) and alfalfa (*M. sativa*). Various leaf forms, such as pinnate, palmate or higher-ordered compound leaf forms, are found in legume species. However, it is not yet known how these different leaf forms are regulated at the molecular level. Studies of compound leaf development in *M. truncatula* not only expand our knowledge of the regulatory mechanisms that underlie compound leaf development in legumes, but also provide the basis for genetic improvements of the yield and quality of legume crops that are an integral part of sustainable agriculture. 

## 7. Conclusions

Studies in tomato, *C. hirsuta* and legumes with compound leaves and *Arabidopsis* and maize with simple leaves unravel that several conserved genetic frameworks are deployed in different morphogenetic processes, including leaf margin serration and patterning during simple and dissected leaf development in diverse species. One is the auxin efflux PIN protein-mediated auxin concentration gradients, or activity maxima, which precede the initiation of leaves from the flanks of the SAM and leaflets from leaf marginal blastozones and serrations from leaf margins. Another one involves the homeodomain KNOXI proteins that promote indeterminacy and the CIN-like TCP protein that promotes leaf maturation. GA and CK act downstream of KNOXI and TCP to fine-tune the morphogenetic activity for compound leaf development. The NAM/CUC proteins, controlling the organ boundary, also play a role in compound leaf development by suppressing the auxin signal between regions of lamina outgrowth. 

Interestingly, SGL1 and PALM1 proteins fine-tune the morphogenetic activity at leaf margins and control compound leaf development in *M. truncatula*, which belongs to the IRLC clade of legumes (Figure 1). In IRLC legumes, KNOXI proteins are not recruited in compound leaf development. However, the processes involving PIN-mediated auxin activity maxima and NAM/CUC proteins are involved in compound leaf development in *M. truncatula*. Intriguingly, a class M KNOX protein with the MEINOX domain, but without the DNA-binding homeodomain, plays a role in trifoliate leaf development. It is foreseeable that future studies in areas of gene regulatory networks, small RNAs and plant growth regulators will expand our knowledge on the origin and evolution of dissected leaves in angiosperm.
